# Clinical and cost effectiveness of a parent mediated intervention to reduce challenging behaviour in pre-schoolers with moderate to severe intellectual disability (EPICC-ID) study protocol: a multi-centre, parallel-group randomised controlled trial

**DOI:** 10.1186/s12888-020-2451-6

**Published:** 2020-01-30

**Authors:** Olayinka Farris, Rachel Royston, Michael Absoud, Gareth Ambler, Jacqueline Barnes, Rachael Hunter, Marinos Kyriakopoulos, Kate Oulton, Eleni Paliokosta, Monica Panca, Laura Paulauskaite, Michaela Poppe, Federico Ricciardi, Aditya Sharma, Vicky Slonims, Una Summerson, Alastair Sutcliffe, Megan Thomas, Angela Hassiotis

**Affiliations:** 1 Division of Psychiatry, University College London, 6th Floor Maple House, 149 Tottenham Court Road, London, W1T 7NF UK; 2 Evelina London Children’s Hospital, St Thomas’ Hospital, Westminster Bridge Road, London SE1 7EH and King’s College London, Strand, London, WC2R 2LS UK; 3 Department of Statistical Science, University College London, Gower Street, London, WC1E 6BT UK; 4 Department of Psychological Sciences, Birbeck University of London, Malet Street, London, WC1E 7HX UK; 5 Research Department of Primary Care and Population Health, Royal Free Medical School, NW3 2PF, London, UK; 6 South London and Maudsley NHS Foundation Trust and Department of Child and Adolescent Psychiatry, Institute of Psychiatry, Psychology and Neuroscience, King’s College London, PO66 De Crespigny Park, London, SE5 8AF UK; 7 Great Ormond Street Hospital for Children NHS Foundation Trust, Great Ormond Street, London, WC1N 3JH UK; 8 The Effra Clinic, 4th Floor, 86-90 Paul Street, London, EC2A 4NE UK; 9 Institute of Neuroscience, Newcastle University, Newcastle upon Tyne, NE1 7RU UK; 10 Contact, 209-211 City Road, EC1V 1JN, London, UK; 11 Institute of Child Health, 30 Guilford Street, London, WC1N 1EH UK; 12 Blackpool Teaching Hospitals NHS Foundation Trust, Blackpool Victoria Hospital, Whinney Heys Road, Blackpool, FY3 8NR UK

**Keywords:** Intellectual disabilities, Challenging behaviour, Randomised control trial, Stepping stones triple P, SSTP, Parenting interventions

## Abstract

**Background:**

Children with intellectual disabilities are likely to present with challenging behaviour. Parent mediated interventions have shown utility in influencing child behaviour, although there is a paucity of UK research into challenging behaviour interventions in this population. NICE guidelines favour Stepping Stones Triple P (SSTP) as a challenging behaviour intervention and this trial aims to evaluate its clinical and cost effectiveness in preschool children with moderate to severe intellectual disabilities.

**Methods:**

This trial launched in 2017 at four sites across England, with the aim of recruiting 258 participants (aged 30–59 months). The Intervention Group receive nine weeks of SSTP parenting therapy (six group sessions and three individualised face to face or telephone sessions) in addition to Treatment as Usual, whilst the Treatment as Usual only group receive other available services in each location. Both study groups undergo the study measurements at baseline and at four and twelve months. Outcome measures include parent reports and structured observations of behaviour. Service use and health related quality of life data will also be collected to carry out a cost effectiveness and utility evaluation.

**Discussion:**

Findings from this study will inform policy regarding interventions for challenging behaviour in young children with moderate to severe intellectual disabilities.

**Trial registration number:**

Clinicaltrials.gov, NCT03086876. Registered 22nd March 2017, https://clinicaltrials.gov/ct2/show/NCT03086876.

## Background

Intellectual Disability (ID) is a lifelong condition characterized by limitations in cognitive ability and adaptive behaviours identifiable from early childhood [1]. A report by the Challenging Behaviour Foundation in 2014 estimated that approximately 10,000 children with ID in the UK show challenging behaviour [2]. Challenging behaviour is defined as actions of such intensity, frequency and duration that it threatens the physical safety of a person or others around them [3]. Behaviours include self-injury, physical aggression and non-person directed behaviour such as property destruction [4]. Whilst challenging behaviour is reported to persist over time, only a small percentage of participants receive intervention [5].

Early intervention has been particularly influential in the improvement of longer term outcomes in children with conduct or behavioural disorders [6–11]. Health Economists have also demonstrated the cost benefit of interventions experienced in the preschool years, given that the early years are the time of maximum brain development, and also of maximum malleability [12–16]. Einfeld and colleagues [17] showed that challenging behaviour increases care costs, which may be prevented with affordable early intervention programmes.

Despite promising evidence from a number of parent mediated interventions with children in the general population (e.g. Sure Start, Video Feedback Sensitive Discipline, and Triple P- Positive Parenting Programme) [18], there is a paucity of UK based early intervention research for reducing challenging behaviours in young children with ID. Most existing interventions have been developed for children with behavioural problems with no specificity to ID.

NICE guidelines (advised by the parent members of the guideline development group) indicated that parenting interventions particularly Stepping Stones Triple P (SSTP), an adapted version of Triple P (TP), demonstrate sufficient evidence in reducing challenging behaviour in children with ID [19]. TP (and SSTP) is a system of psycho-educational and behavioural approaches that aim to increase parental confidence and skills so that parents are able to manage the child’s behaviour effectively. SSTP comprises different levels depending on increasing family complexity, with Level 4 recommended for parents of children with severe behavioural problems. Efficacy trials outside the UK have indicated significant reductions in challenging behaviour in children with ID [20]. Although there is a scarcity of economic data for SSTP itself, trial and observational data from a number of countries suggest delivery of TP may be cost-effective, especially if it were applied at population levels [21, 22].

### Aims

This study aims to evaluate the clinical and cost effectiveness of Level 4 SSTP in a multi-site parallel cluster randomised control trial of preschool children with moderate to severe ID 12 months post randomisation. Treatment as usual (TAU) is available to participants in both arms of the trial.

The primary hypothesis is that the addition of level 4 SSTP to TAU will reduce challenging behaviour on completion of the intervention at 12 months post randomisation, compared to TAU alone. The primary outcome is the severity of challenging behaviour using the parent completed preschool Child Behaviour CheckList (CBCL) [23].

The secondary hypotheses include:

- SSTP will reduce challenging behaviour measured at 12 months post randomisation in blind rated observations and caregiver/teacher questionnaire measures.
- SSTP will be more cost-effective compared to TAU.

## Method

### Trial design and setting

**Fig. 1:**
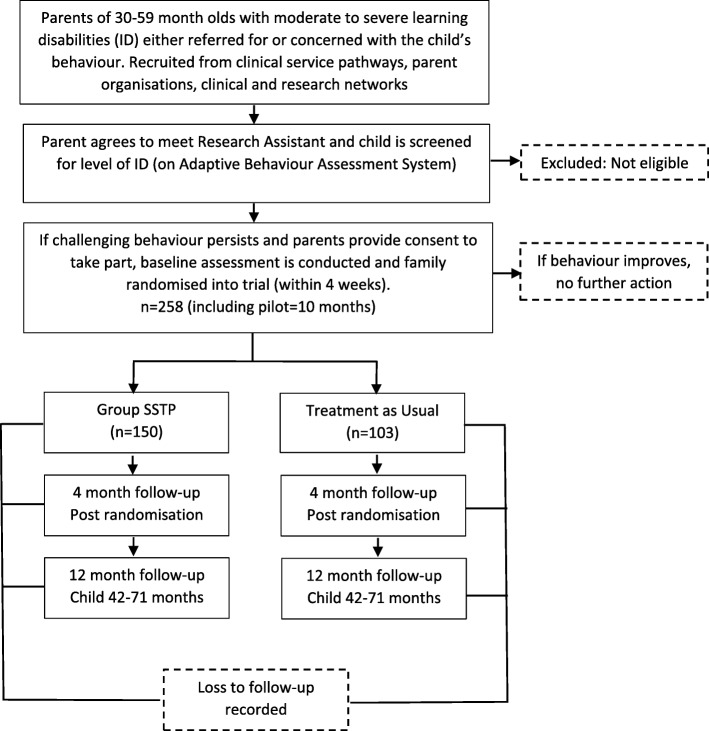
Flow chart of the study design. This chart demonstrates the flow of participants through the trial from initial contact to the completion of the follow-up assessments

The randomised control trial is parallel and two-armed with blinding of outcome assessors. It includes a process evaluation with parent qualitative interviews to enhance understanding of the appropriateness and feasibility of the intervention. The study was planned and implemented in accordance with the Consolidated Standards of Reporting Trials (CONSORT) extension standards to compare the cost-effectiveness of the combination of SSTP plus TAU, versus TAU alone in reducing challenging behaviour at 12 months post randomisation. The trial design is summarised in Fig. 1.

Study participants are recruited from a wide variety of services within the participating centres in North and South London, North East (Newcastle and surrounding areas) and North West England (Blackpool and surrounding areas). Services include NHS settings, e.g. Child Development Teams; Child and Adolescent Mental Health Services; education (nursery/preschool) and third sector organisations e.g. caregiver groups. A number of Participant Identification Centres have also been opened (Table 1).

**Table 1:** List of recruitment sites and participant identification centres

Site	Participant Identification Centres (PICs)
Blackpool Teaching Hospitals NHS Foundation Trust	None
Central and North West London NHS Foundation Trust	Royal Free London NHS Foundation Trust
	Imperial College Healthcare NHS Trust
	Great Ormond Street Hospital NHS Foundation Trust
	Primary Care (Islington/Camden GP practices, Barnet CCG practices, Enfield CCG practices)
Guy's and St Thomas' NHS Foundation Trust	St George’s University Hospital NHS Foundation Trust
	Lewisham and Greenwich NHS Trust
Cumbria, Northumberland, Tyne and Wear NHS Foundation Trust	Northumbria Healthcare NHS Foundation Trust
	South Tyneside and Sunderland NHS Foundation Trust
	Newcastle upon Tyne Hospitals NHS Foundation Trust
	Gateshead NHS Foundation Trust

**Note:** *CCG* Clinical Commissioning Groups

#### Eligibility criteria

Parents of young children concerned about their child’s behaviour within the four participating centres are eligible for inclusion in the study if:

1. Parents are at least 18 years of age.
2. Child is aged 30–59 months at identification.
3. Child has moderate to severe ID (parent reported Adaptive Behaviour Assessment System (ABAS) General Adaptive Functioning score of 40–69) [24].
4. Reports of challenging behaviour over a 6- month period but no less than 2 months.

Exclusion Criteria

1. Child has mild, profound or no ID on parent reported ABAS.
2. Parent/carer has insufficient English language to complete study questionnaires.
3. Another sibling is taking part in the study.

#### Participant identification and screening

Eligible participants are identified by the community paediatric and child and adolescent mental health teams in each of the four areas. Health or social care professionals identify eligible participants through new referrals or existing cases. Identification involves reviewing or screening identifiable personal information of participants by members of the regular clinical team. A member of clinical staff/clinical study officer contacts eligible participants, gives an introduction to the study and the study Patient Information Sheet. All participants who are interested in taking part complete an Expression of Interest form which is then passed on to the researchers.

Parents need to consent to the screening process, including the parent administered ABAS about the child’s adaptive behaviour/level of functioning. This determines whether a child’s level of intellectual ability falls within the inclusion criteria, and confirms that the child has had challenging behaviours continually in the past two months, i.e. such behaviours being present several times a week. Where the child fulfils the adaptive function range, the baseline assessment (assuming consent is given) takes place following which the participant is randomised either to the intervention arm (SSTP plus TAU) or TAU.

#### Interventions

##### SSTP plus TAU

Level 4 Stepping Stones Triple P is a 9 week psycho-education programme with 6 group sessions and three individual telephone or face to face contact with participants. SSTP is an adapted parenting programme for children with ID. The sessions cover strategies that parents can use to reduce unwanted behaviours, maintain behavioural change, cultivate a positive relationship with their child and facilitate independent problem solving. Each group session lasts approximately 2.5 h and individual sessions last about 30 min. Parents receive a course book with topics to be covered in each session and are contacted by the therapist if a session is missed.

##### Treatment fidelity

Each therapist (eight: two per centre) responsible for delivering SSTP has been trained in the Stepping Stones Training and Accreditation programme. Therapists were observed by TP trainers to build therapist competence and are provided with monthly supervision to ensure fidelity of the intervention and ongoing support and skills maintenance as is appropriate for psychosocial interventions [25]. Further, to determine whether treatment was delivered as intended (adherence), each therapist completes individual session checklists and all the sessions are videotaped to be rated by independent assessors (competence). A random 10% of assessments is double rated for reliability by an external blinded expert. Therapist deviations from the manualised intervention will be recorded to examine where flexibility may be required based on individual participant needs.

##### Treatment as usual

Parents continue to access interventions and therapies on offer to them in their local area, including a range of services such as support from health visitors, primary care engagement and advice, early intervention provided by community paediatric services or Child and Adolescent Mental Health Services, and parenting advice and support sessions by carers’ groups or other third sector organisations. It includes evidence based treatments (e.g. Webster Stratton Incredible Years, Early Bird and Strengthening Families interventions), support groups by third sector and voluntary groups (e.g. the charity Contact and locally organised parent groups) and via the NHS, which provides psychological and behavioural therapies as part of professional care (please see Additional File 1 for a more comprehensive list of available interventions). Parents allocated to both arms of the trial also receive a list of national resources and the Contact guide to challenging behaviour with tips and advice on social and health care supports.

#### Outcome measures

Outcomes are measured by self-report, direct parent-child observations and face-to-face interviews (See Table 2 for list of measures). All participants are assessed at the following time points: screening (T1), baseline (T2), four months post-randomisation (T3) and 12 months post-randomisation (T4). The assessment window for follow-ups is four weeks. Data collected outside these time windows will be recorded but not used for the main analyses (see Table 3 for the schedule of assessments at each time point).

**Table 2:** List of study outcome measures

Outcome	Measure details
Primary outcome measure: Child Behaviour Checklist (CBCL)	Each question relates to a specific behaviour and is measured on a 3-point Likert Scale. Overall scores are derived for behavioural difficulties, attention problems and aggression. A T-score of Total Problem Behaviours of 60 or over signifies borderline to clinical caseness. CBCL incorporates DSM-5 diagnostic categories which rate comorbidities, e.g. autism spectrum disorders, mood disorders.
Mullen Scales of Early Learning	Assesses child level of disability [26]. Only assessed during the baseline assessment.
Revised Family Observation Schedule (FOS-RIII)	FOS-RIII is an objective measure of parent-child interaction, previously used in studies investigating SSTP and codes 20 min (four 5-min consecutive sections) home based videotaped parent-child interactions [27]. There will be an inter-rater reliability exercise on a proportion of the observations to ensure reliability.
Child Behaviour Checklist Caregiver-Teacher Report Forms (C-TRF)	Most children in the sample age range will have additional care outside the parental home allowing us to have additional perspectives on the child’s behaviour. The CBCL and C-TRF are extensively used to measure child’s behaviour and there are positive reports about high completion rates by teachers/nursery staff as shown in other studies [28].
General Health Questionnaire (GHQ)	Common psychiatric morbidity in the parent will be assessed at baseline, 4-month and 12-month follow-up.
Questionnaire on Resources and Stress (QRS-F short form)	Measures parental stress in caregivers of chronically ill or children with ID at baseline, 4-month and 12-month follow-up [29].
Caregiving Problem Checklist-Difficult Child Behaviour	The frequency of difficult child behaviour when the parent is completing care-giving tasks will be measured at baseline, 4-month and 12-month follow-up. Internal consistency is adequate (α = .78) [30].
Parenting Sense of Competence Scale (PSOC)	Assesses Satisfaction and Efficacy competencies as a parent at baseline, 4-month and 12-month follow-up [31]. Internal consistency for the measure ranges from α = .70–.80 [31].
Child and Adolescent Service Use Schedule (CA-SUS)	A modified version will be used in the trial to assess child health and social care service use at baseline, 4-month and 12-month follow-up [32].
Paediatric Quality of Life (PedsQL)	Assesses health related quality of life. The measure covers Physical, Emotional, Social, and School Functioning domains. It contains a parent proxy report for children aged 2 years and over and will be used in the study to derive Quality-Adjusted Life Year (QALYS) for the health economic evaluation. Internal consistency for the parent version is acceptable (α = .86) and has demonstrat4ed discriminant validity. This will be completed at baseline, 4-month and 12-month follow-up [33].
EuroQol-5D	Captures parental and caregiver perspective on their health status at baseline, 4-month and 12-month follow-up which will be used in the economic evaluation [34].
Client Satisfaction Questionnaire	Measures parent intervention acceptability [35]. The questionnaire will allow parents to provide feedback about the intervention during the 4 month follow-up by commenting on their satisfaction with and experience of the intervention, including ease of use, format and helpfulness. It has been specifically developed for research in SSTP and has high internal consistency (α = .92) [30].
Case Report Forms (CRF)	To collect sociodemographic and clinical information about comorbidities.

**Table 3:** Schedule of assessments

Visit no	1	2	3	4
Tasks	Screening	Baseline assessment*	4 month follow-up	12 month follow-up
Allowed deviation window	n/a	+/ week	+/− 4 weeks	+/− 4 weeks
Informed consent (screening)	x			
Assessment of eligibility criteria	x	x		
Adaptive Behaviour Assessment Schedule (< 69)	x			
Research assessments minimum 1 week, maximum 4 weeks after screening)				
Informed consent (research)		x		
Mullen Scales of Early Learning		x		
CRF		x		
Preschool CBCL		x	x	x
Parent-child observation and FOS-RIII		x	x	x
C-TRF		x	x	x
GHQ-12		x	x	x
QRS-F short form		x	x	x
Caregiving Problem Checklist-Difficult Child Behaviour scale		x	x	x
PSOC		x	x	x
CA-SUS		x	x	x
Client Satisfaction Questionnaire			x	
Peds-QL		x	x	x
EQ-5D		x	x	x

*: at baseline, all assessments will be carried out prior to randomisation

The primary outcome measure is the parent completed Child Behaviour Checklist (CBCL; 24) at 12 months. The CBCL is a robust and widely used questionnaire which measures child behaviour and has been previously used in clinical trials and epidemiological studies of children with ID [36, 37]. Secondary outcomes include direct observations of parent-child interactions, parental health, stress and competence, service use and health related quality of life (Table 2).

### Sample size

A sample of 258 children (SSTP: 155 children, TAU: 103 children) will allow us to detect a low to moderate (standardised) effect size of 0.40 for the primary outcome at the 5% significance level, with 90% power. This is equivalent to detecting a clinically meaningful difference between the two treatment groups of 8 points, assuming a standard deviation of 20. This calculation is based on baseline-adjusted (ANCOVA) analysis assuming a correlation of 0.5 between baseline and follow-up measurements. In addition, the calculation has been adjusted for therapist clustering, assuming an intra-class correlation of 0.05, average group size of 7, and an anticipated drop-out of 10%.

### Recruitment

It was estimated that 22 months would be needed to recruit the total sample of 258 at a recruitment rate of 12 children per month. Such rates are similar to other studies of children with neurodisability [38]. Data from the participating sites suggest they receive in excess of 100 referrals a year (and as many as 300), at least a third of whom could be eligible for the study. Therefore, recruitment of the required number of participants within this age range was deemed feasible. A multisource referral strategy is being followed, facilitated by the clinical research networks, national, clinical and third sector contacts, as well as social media. Participants receive £15 vouchers at each assessment time point for time donated to the study. In addition, child care and travel for participants may also be reimbursed where appropriate.

### Methods – assignment of interventions

#### Allocation

Participants are randomised using a 3:2 allocation ratio to either SSTP or TAU using randomly permuted blocks of varying block sizes and stratification by site and level of ID (moderate and severe). Randomisation and data management is provided by Sealed Envelope [39], a commercial clinical randomisation and data management web service.

#### Allocation concealment and implementation

Research assistants enter baseline assessment results on a web-based case report form (CRF). Each case is assigned a study number and parents and therapists are given information about allocation status. Arrangements are then made to commence the group sessions. Researchers are in separate departments than staff involved in delivery of level 4 SSTP. The therapists do not treat any families allocated to TAU.

#### Blinding

Although it is not possible to blind trial participants or therapists delivering the intervention, parents are reminded not to disclose any details about their treatment to the research team during assessments. Research assistants and the lead statistician remain blind to trial arm allocation, with the lead statistician not attending the closed part of the Data Safety and Monitoring Board (DSMB) meeting. Any violations of the study protocol are recorded and reported to the Trial Steering Committee and the DSMB.

#### Data collection methods

All data are collected and handled in accordance with PRIMENT Standard Operating Procedures (SOPs). A unique identification number is assigned to each participant and all identifying participant information is stored separately and securely in UCL Data Safe Haven, a secure system for storing sensitive study information. Source data verification checks will be completed on 100% of the primary outcome measure, as well as for 5% of all secondary outcome measures. The delegation log identifies all those personnel with responsibilities for data collection and handling, including those who have access to the trial database.

Long-term experience of studies with people with ID suggests that very few are lost to follow up (e.g. PBS study, TIME-A study). Participants may still wish to meet with the researchers but not take part in interventions. Therefore, although we shall stress that participants can withdraw at any time without giving a reason, we shall retain any assessment records that have been carried out to that point and we shall maintain contact unless told otherwise.

#### Data management

Audio and video recordings are stored on Data Safe Haven and are deleted from the digital machines from which they were originally recorded. The CRFs are entered into a web-based clinical data management system, Red Pill, provided by Sealed Envelope through PRIMENT. Original copies of outcome measures are stored in locked cabinets in a locked office. At the end of the trial, prior to analysis, PRIMENT SOP Database Lock, Unlock and Closure will be followed. All aspects of data management of the study comply with the UK Data Protection Act 1998, PRIMENT SOPs, GDPR and Good Clinical Practice.

#### Patient and public involvement

Parents of children with ID and challenging behaviour from the Camden Special Needs Forum assisted in the development of the study proposal. Four parent members of the national charity CONTACT were also recruited to form the Parent Advisory Group (PAG), meeting four times annually to assist in overseeing the trial, discussing study progress and helping with materials. The PAG will be involved with reviewing the full study report and dissemination plan.

#### Process evaluation

To understand how psychosocial interventions work in practice, particularly due to the paucity of SSTP delivery within the UK NHS, a process evaluation utilising a mixed methods approach has been included within the trial. This includes assessment of what is delivered (fidelity, dose, adaptations, reach), collection of the opinions of a stratified purposive stakeholder sample, i.e. participants (those in the intervention/TAU groups, as well as those who have declined, approximately 10–12 from each); 6–8 service managers; and all therapists. Parents participating in these qualitative interviews receive an additional £15 voucher. Therapists also ask parents in the intervention arm to complete a brief satisfaction questionnaire to assess satisfaction with the intervention at the end of the final group session.

### Statistical methods

#### Analysis of primary outcome

A consort flow diagram [40] is used to describe the progress of participants through the study and the follow-up at different time points. Descriptive analyses (means, standard deviations, relevant quantiles and proportions) will be used to summarise the characteristics of the children in each study arm. A comparison of baseline characteristics will be performed to assess whether balance has been achieved; any notable imbalances may lead to additional adjusted analyses for continuous outcomes.

The primary analysis of the CBCL score at 12 months will use mixed models to perform an individual level analysis and will follow Roberts and Roberts (2005) [41] in adjusting for therapist clustering in the intervention arm only (random coefficient model). The final model will also adjust for baseline CBCL score and randomization stratification factors (centre, level of ID) using fixed effects. All modelling assumptions will be checked and a sensitivity analysis will be performed relaxing the heteroscedasticity assumption. Significance will be considered at the 5% level and confidence intervals with be at the 95% level.

Additional analyses will be performed for the secondary outcomes. Continuous outcomes will be analysed using a similar modelling approach to that described for the primary outcome, but for binary outcomes we shall use logistic mixed models [42]. The amount of missing data in each trial arm will be reported and we will investigate its impact on the balance achieved by randomisation. We will also explore whether missingness is associated with any participant characteristics, using descriptive comparisons and tests as appropriate. This may lead to further adjusted analyses. Stata 15 and R 3.6.0 or above will be used to perform the analyses.

#### Economic evaluation

The economic evaluation will be conducted from a health and social care perspective in the primary analysis, and from a societal perspective in a secondary analysis, which includes the impact on quality of life of parents and other caregivers contributing to the child’s care. The Child and Adolescent Service Use Schedule (CA-SUS) [43] tracks the personal, societal and health service resource usage in the past 6 months at baseline and 12-month follow-up and in the past 4 months at 4-month follow-up. The primary analysis will include only health and social care data collected as part of the trial, meaning this will only cover 10 months of the trial (missing months 4 to 6). Sensitivity analyses will be used to project costs from the follow-ups to estimate the 12-month health and social care resource use. UK unit costs obtained from publicly available sources will be applied to each resource item in both arms of the trial [44, 45]. Benefits payments will be costed from government statistics. Data on delivery of the intervention will be collected to calculate the cost of the intervention using micro-costing methods [46].

The overall economic evaluation will comprise: 1) Cost-effectiveness analysis estimating the mean incremental cost per change in CBCL; 2) Exploratory analysis of quality of life using the PedsQL to predict utility scores. There is no single, valid, preference-based measure for health state valuation in children under the age of 5 or children with ID and therefore it is not currently possible to calculate Quality-Adjusted Life Years (QALYs) for use in cost-utility analysis [47]. Therefore, the PedsOL and the mapped EQ-5D-Y utility scores algorithm [48, 49] will be utilised to calculate QALYs; 3). Cost-benefit analysis of the impact on the parents/caregivers.

Cost and effect data will be combined to calculate incremental cost-effectiveness ratios for each analysis. We shall use non-parametric bootstrap estimation to derive 95% confidence intervals for mean cost and effect differences between the trial groups and to calculate 95% confidence intervals around the incremental cost-effectiveness ratios. We will include adjustments for baseline values (costs, CBCL and effects) in the three analyses. Cost-effectiveness acceptability curves, showing the percentage of cases for which, the intervention is cost-effective will be constructed using the bootstrap data. A series of sensitivity analyses will be undertaken to explore the implications of uncertainty on the incremental cost-effectiveness ratios.

#### Data monitoring

The study is overseen by a Data Safety and Monitoring Board (DSMB) which has six monthly meetings to manage trial procedures. Members have no competing interests and are independent from the study sponsor and organisers. Interim analyses are supplied to the DSMB which advise the Trial Steering Committee (TSC) on whether the active intervention is successful and whether the economic outcomes evidence is sufficient to guide recommendations for SSTP to health care providers. No interim analyses are planned.

#### Harms

Serious Adverse Events (SAE; untoward occurrences that result in harm) are reported via the eCRF by the trial manager within 24 h of becoming aware of the event. All reports are reviewed by the CI or PIs within 2 days of receiving the report and the outcome is recorded in the eCRF. SAEs that are determined to be related and unexpected are reported to the ethics committee and to PRIMENT, in accordance with the PRIMENT non-clinical trial of an investigational medicinal product (Non-CTIMP) safety management SOP.

#### Auditing

Site visits are conducted at all sites annually. The site file, consent forms, enrolment and screening logs are checked and source data verification checks are conducted. A monitoring report is usually compiled and reviewed by PRIMENT.

#### Confidentiality

All study-related information is stored securely at the study site. All participant information is stored in locked file cabinets in locked rooms with limited access and data is also stored through the secure online system, Data Safe Haven. Data is identified by identification numbers to maintain participant confidentiality. Personal information is stored separately from the study records.

#### Dissemination policy

The study papers will be published in high impact journals and targeted communications for parents will be published through the charity Contact. Contact will also advise on other media and policy opportunities that allow for dissemination. Findings will be communicated at local, national and international conferences including for lay and parent groups. A study report for the funders will be posted on the HTA website. Parents will be involved at all stages and take part in commenting on reports and papers prior to publication as well as leading on presentations. In the event of a negative trial, it is important to know whether treatment as usual as currently provided in the services in England may be as effective as a manualised intervention. Therefore, the study, regardless of outcome, will be published and disseminated. All co-applicants will be listed as authors. The protocol will be published in an open access journal; the dataset and statistical codes will be available by sharing agreements upon request.

#### Department of Health Disclaimer

This report presents independent research commissioned by the National Institute for Health Research (NIHR). The views and opinions expressed by authors in this publication are those of the authors and do not necessarily reflect those of the NHS, the NIHR, MRC, CCF, NETSCC or the Department of Health.

#### Trial status

Participant recruitment in this study commenced in September 2017. A 10-month internal pilot tested feasibility, acceptability and recruitment at all sites, using the full baseline and eligibility and follow-up assessment battery for all cases and the start-up of SSTP delivery to some of the participants. The progression criterion was that the rate of recruitment between months 5–14 should be at least 70% of the rate expected (no fewer than 8 children per month). This was achieved and the Trial Steering Committee advised NIHR to proceed to the full trial. The study is actively recruiting. To date, 190 participants have been randomised into the study and 19 groups have been completed.

## Discussion

This paper describes the study protocol for a multicentre trial designed to investigate the clinical and cost-effectiveness of a parent mediated intervention (SSTP) to reduce challenging behaviour in pre-schoolers with moderate to severe ID. Challenging behaviour is prevalent and persistent in children with moderate to severe ID and is associated with many negative outcomes, including later psychiatric morbidity [4, 50–52], poorer quality of life, parental stress and high service costs [53–56].

SSTP is a parenting programme that has demonstrated efficacy in non-UK randomised controlled trials to reduce challenging behaviour in children and improve parent outcomes [19, 57, 58]. A recent meta-analysis of 16 studies has highlighted the large evidence base for Level 4 SSTP, which had the strongest treatment effects compared to other SSTP levels for improving behaviour in children with ID [20]. However, the majority of studies included small sample sizes (mean = 60.2, SD = 43.8) and randomised control trials of SSTP have not been conducted in the UK to compare this intervention to other readily available therapies.

To our knowledge, this is the first UK trial to evaluate the effectiveness of SSTP as a parent mediated intervention for challenging behaviour in very young children. The findings will inform real-world practice and NICE clinical recommendations about the provision of group interventions for children with moderate to severe ID [51]. We have already identified significant variability in available services for this population across the 4 sites in the UK and therefore, rolling out the intervention should be underpinned by appropriate evidence.

The study has several strengths, it is multicentre and is powered to detect a significant difference between intervention and TAU arms. The primary study outcome measure (CBCL) is a robust and widely validated measure of behaviour, which will be further validated through the use of behavioural observations of challenging behaviour at each time point. Additionally, data from multiple respondents is being collected on the CBCL (e.g. parents and another caregiver such as a teacher) to examine accuracy and enable the potential measurement and comparison of behaviours across different contexts and with different caregivers. The secondary outcome measures will also provide critical information on variables that are known to impact child behaviour [59, 60]. Moreover, conducting a process evaluation and fidelity testing ensures the external validity of the study and will examine gaps in the implementation that are likely to hinder uptake [61].

Limitations include the lack of blinding of participants, potential attrition of participants and deviations in the delivery of the intervention. Further, only parents with sufficient English to respond to the questionnaires and follow the intervention guidance can be recruited into the study.

In conclusion, the results of the trial described in this protocol will provide a vital contribution to intervention research for reducing challenging behaviour in children with ID. This population is said to be underserved, with almost no access to evidence based interventions for challenging behaviour early in life [62]. The trial will inform policy on the clinical and cost-effectiveness of SSTP, with the aim of improving behaviour and outcomes for children with moderate to severe ID and their families.

## Supplementary information

**Additional file 1. ***List of known interventions/therapies on offer at participating sites*. This additional file provides a list of examples of some known interventions/therapies available at participating sites. All participants in the study (intervention and treatment as usual) can attend any additional therapies during participation in the study. This list is indicative of the variety of interventions on offer (as of August 2019) at participating sites and is not exhaustive.

## Data Availability

The datasets generated during the current study are not publicly available as data collection is ongoing. Data will be available to Principal Investigators on request once the dataset is clean and locked and the study publications completed.

## Abbreviations

ABAS: Adaptive Behaviour Assessment System
CA-SUS: Child and Adolescent Service Use Schedule
CBCL: Child Behaviour Checklist
CI: Chief Investigator
CRF: Case Report Form
CTIMP: Clinical Trial of an Investigational Medicinal Product
C-TRF: Child Behaviour Checklist Caregiver - Teacher Report Forms
DSM-5: Diagnostic and Statistical Manual of Mental Disorders 5th Edition American Psychiatric Association
DSMB: Data Safety and Monitoring Board
EQ-5D: EuroQoL Five Dimensions Scale
FOS: Revised Family Observation Schedule, FOS-RIII
GCP: Good Clinical Practice
GDPR: General Data Protection Regulation
GHQ: General Health Questionnaire
GP: General Practitioner
HRA: Health Research Authority
ID: Intellectual Disability
Main REC: Main Research Ethics Committee
NHS R&D: National Health Service Research & Development
NIHR HTA: National Institute for Health Research Health Technology Assessment Programme
PAG: Parent Advisory Group
QALYs: Quality-Adjusted Life Years
SOP: Standard Operating Procedure
SSTP: Stepping Stones Triple P
TAU: Treatment as Usual
Term: Definition
TMG: Trial Management Group
TP: Triple P
TSC: Trial Steering Committee
