# Supplementary material for: Clinical and cost effectiveness of a parent mediated intervention to reduce challenging behaviour in pre-schoolers with moderate to severe intellectual disability (EPICC-ID) study protocol: a multi-centre, parallel-group randomised controlled trial
Source: BMC Psychiatry. 2020 Jan 30;20:35. doi: 10.1186/s12888-020-2451-6 (PMC6993328; doi:10.1186/s12888-020-2451-6)
Supplement: Supplementary file 1 — Additional file 1. List of known interventions/therapies on offer at participating sites. This additional file provides a list of examples of some known interventions/therapies available at participating sites. All participants in the study (intervention and treatment as usual) can attend any additional therapies during participation in the study. This list is indicative of the variety of interventions on offer (as of August 2019) at participating sites and is not exhaustive. [file 12888_2020_2451_MOESM1_ESM.docx]

**Additional File 1**

This supplementary materials provides a list of examples of some known interventions/therapies available at participating sites. All participants in the study (intervention and treatment as usual) can attend any additional therapies during participation in the study. Only participants in the intervention arm of the study can attend Stepping Stones Triple P.

Please note this list is indicative of the variety of interventions on offer (as of August 2019) at participating sites and is not exhaustive.

**NHS Child and Family Services**

- Barnet Community Paediatric Service
- Blenheim Child Development Centre, Blackpool
- Camden MOSAIC
- Cheviots Children’s Disability Centre, Enfield
- Community Learning Disability Service, Northumberland and North Tyneside
- Early Years Mental Health Service, Sunderland
- Kaleidoscope Children’s Centre, Lewisham
- SLaM Child and Family Service, Southwark

**Parenting courses**

- Beautiful babies, beautiful brains
- Being a Parent
- Cygnet Parent Programme
- Early Help
- Early Years
- Family Links
- Happy Parents Happy Families
- Making a Positive Start for Autism (MAPS)
- More than Words (The Hanen Program)
- My C.H.I.L.D
- Positive Parenting Skills
- Stepping Stones Triple P
- Strengthening Families Strengthening Communities
- The Nurturing Programme
- Triple P
- Webster Stratton Incredible Years

**Voluntary and third sector services**

Locally organised carer support groups

- Bishop Isaac Community Group - Bexley
- Families 1^st^ 4 Additional Needs [F.F.A.N] – Newcastle
- Gateshead Autism Group
- In it Together - Northumberland
- Pass it on to Parents – Newcastle
- Our Voice – Enfield

Services and support offered by local/regional charities

- Aiming Higher – Blackpool
- Autism Northumberland
- Better Start Blackpool
- Blackpool Carers Centre (Hand in Hand project)
- Camden Special Parents forum
- Enfield Carers Centre
- Little Treasures Autism Charity North East
- South London Family Centre
- Skills for People – Newcastle
- Sunderland Carers Centre
- The KAYAKS – South Tyneside
- Toby Henderson Trust – North East
- WECAN – Northumberland

Services and support offered by national charities

- Council for Disabled Children
- DAZU
- Early Bird – National Autistic Society
- Early Intervention Support Service
- Family Lives
- Home Start
- Mencap
- Norwood
